# Supplementary material for: Tumor microenvironment remodeling plus immunotherapy could be used in mesenchymal-like tumor with high tumor residual and drug resistant rate
Source: Commun Biol. 2023 Dec 18;6:1281. doi: 10.1038/s42003-023-05667-4 (PMC10728080; doi:10.1038/s42003-023-05667-4)

Supplementary figures and supplementary figure captions

Tumor microenvironment remodeling plus immunotherapy could be used in mesenchymal-like tumor with high tumor residual and drug resistant rate

Authors and affiliations

Shuai Shen[1#], Xing Liu[1#], Qing Guo[1#], Qingyu Liang[2] Jianqi Wu[1], Gefei Guan[2], Cunyi Zou[2], Chen Zhu[2], Zihao Yan[2], Tianqi Liu[1], Ling Chen[3], Peng Cheng[2*], Wen Cheng[1*], Anhua Wu[1*].

1 Department of Neurosurgery, Shengjing Hospital of China Medical University, Shenyang, Liaoning, China

2 Department of Neurosurgery, The First Hospital of China Medical University, Shenyang, Liaoning, China;

3 Department of Neurosurgery, Chinese People's Liberation Army of China (PLA) General Hospital, Medical School of Chinese PLA, Institute of Neurosurgery of Chinese PLA, Beijing, China;

# These authors contributing equally.

*** Corresponding Authors:**

An-Hua Wu

Department of Neurosurgery, Shengjing Hospital of China Medical University, No.36 Sanhao Road, Shenyang 110004, China.

Phone: 86-13940166906;

E-mail: ahwu@cmu.edu.cn

Wen Cheng

Department of Neurosurgery, Shengjing Hospital of China Medical University, No.36 Sanhao Road, Shenyang 110004, China.

Phone: 86-15040235535;

E-mail: cmu071207@163.com

Peng Cheng

Department of Neurosurgery, The First Hospital of China Medical University, Shenyang, Liaoning, China.

Phone: 86-13898109126

E-mail: chengpengcmu@cmu.edu.cn

Supplementary Figure 1 EMTCGs could be used to identify mesenchymal subtype tumors.

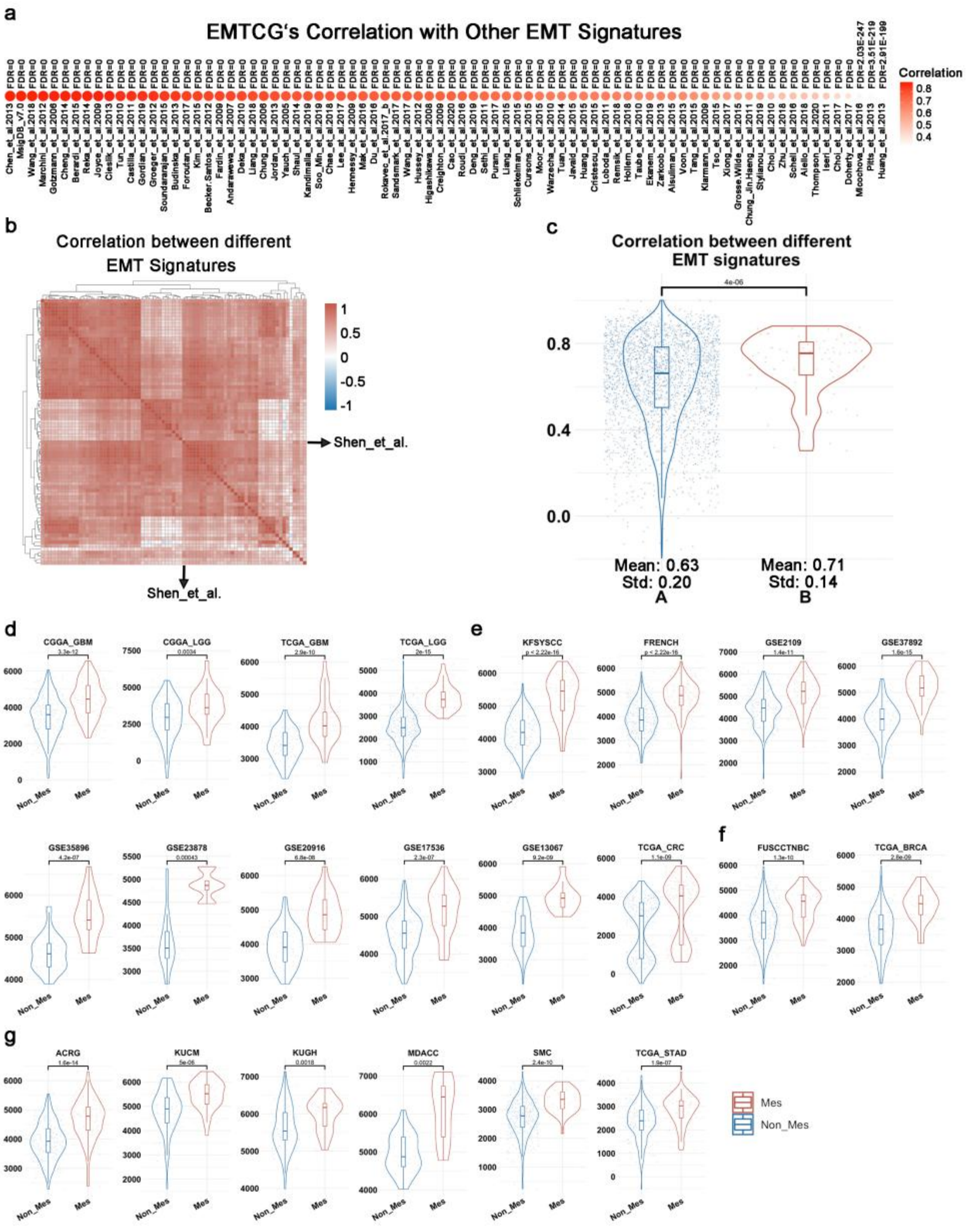

a. EMTCG signature shown highly positive correlation with other EMT related signatures.

b. Each EMT related signature shown positive correlation with other EMT related signatures.

c. The correlation (mean correlation co-efficiency = 0.71, Std = 0.14) between EMTCG signature with other 76 signatures is stronger than the correlation (mean correlation co-efficiency = 0.63, Std = 0.20) among each two signatures of other 76 already published signatures; Group A: correlation among each two signatures of 76 other published signatures; Group B: correlation between our EMTCG signature with other 76 published signatures.

d. Comparison of EMTCGs score between mesenchymal like and non-mesenchymal like tumors in four glioma cohorts. EMTCGs score was upregulated in mesenchymal like gliomas.
e. Comparison of EMTCGs score between mesenchymal like and non-mesenchymal like tumors in 10 colorectal cancer cohorts. EMTCGs score was upregulated in mesenchymal like colorectal cancers.
f. Comparison of EMTCGs score between mesenchymal like and non-mesenchymal like tumors in two breast cancer cohorts. EMTCGs score was upregulated in mesenchymal like breast cancers.
g. Comparison of EMTCGs score between mesenchymal like and non-mesenchymal like tumors in six gastric cancer cohorts. EMTCG score was upregulated in mesenchymal like gastric cancers.
Student's t test was used to analyze statistical significance between 2 groups.

Supplementary Figure 2 EMTCGs were dysregulated in epithelial cancers due to multi-omics mechanisms

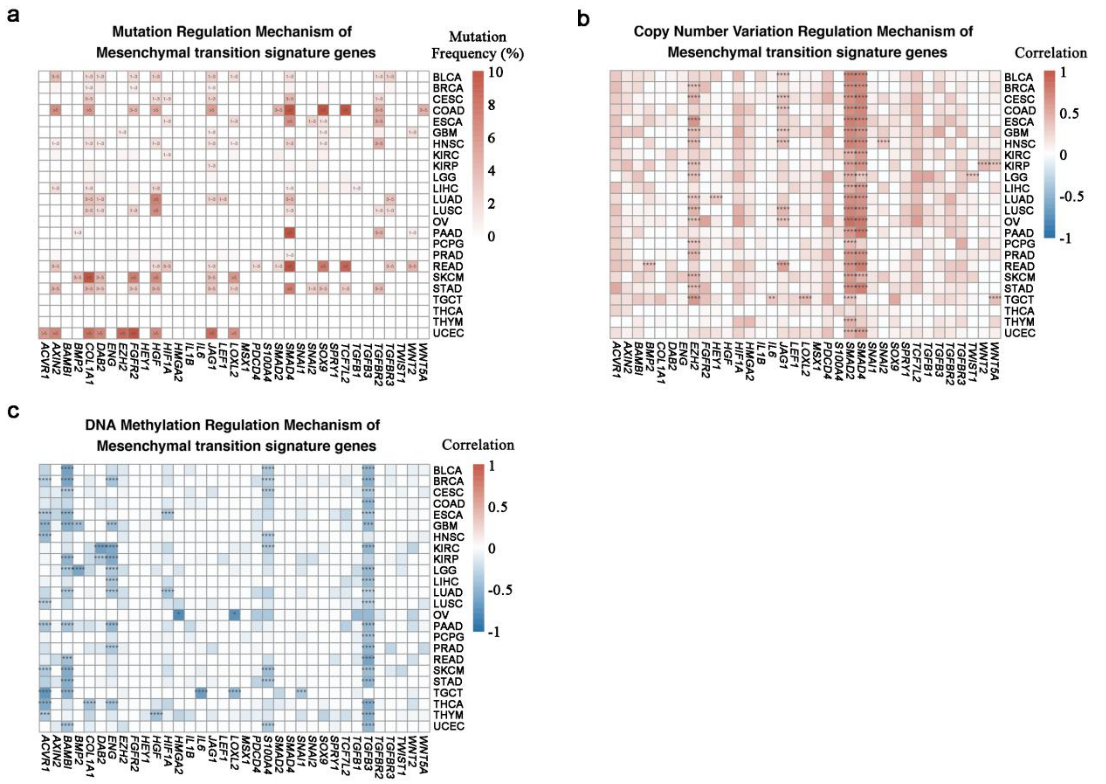

a. Mutation-based expressional regulation mechanism of EMTCGs. On the heatmap, the square cell color depicts the mutation frequency of EMTCGs. Mutation frequency >3% and fold change >1.5 were considered as significant.

b. Correlation analysis of EMTCGs mRNA expression with copy number variation. ****FDR < 0.0001; **FDR < 0.01.

c. Correlation analysis of EMTCGs mRNA expression with DNA methylation probe. ****FDR < 0.0001; ***FDR < 0.001; *FDR < 0.05.

Supplementary Figure 3 Mesenchymal transition was a poor prognosis indicator

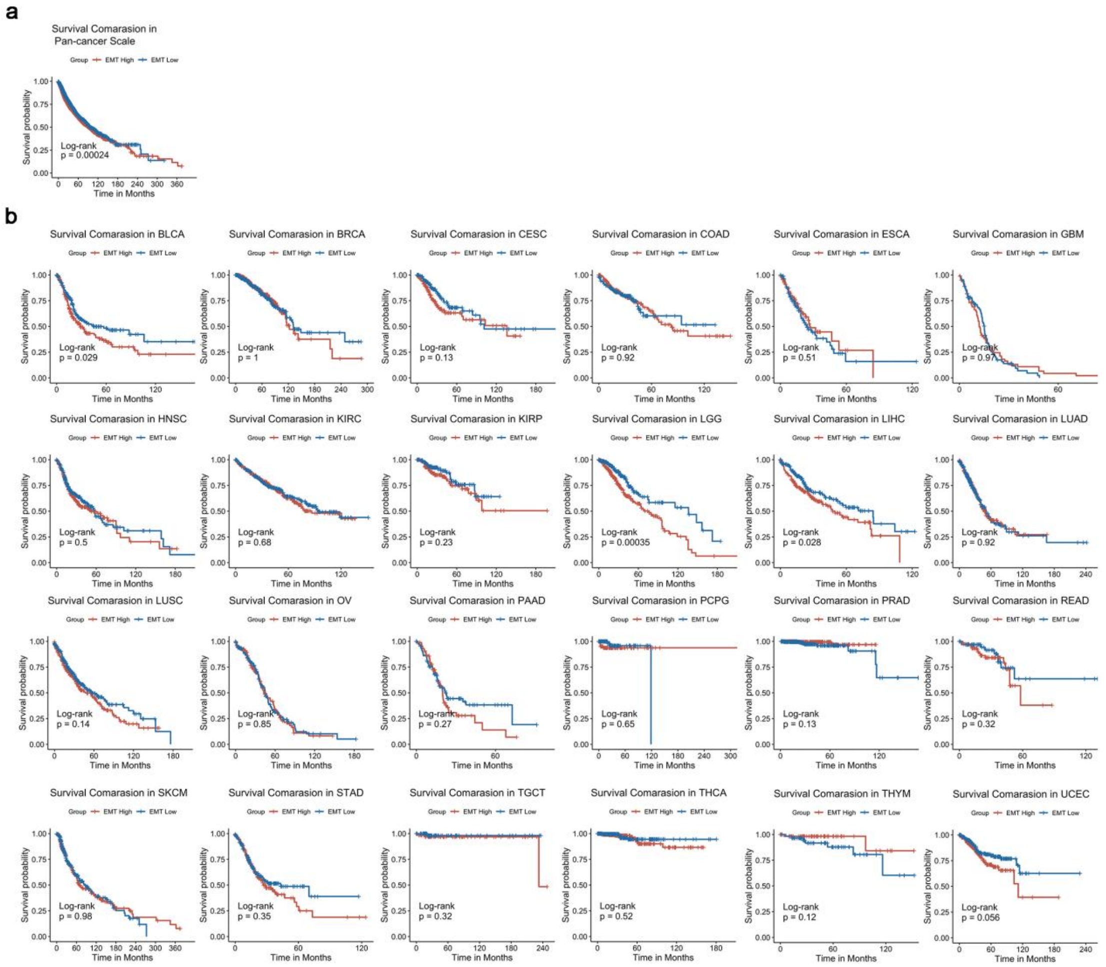

a. Kaplan–Meier survival analysis of overall survival of 9415 samples across 24 cancer types based on the EMTCG score in TCGA cohort. The red and blue curves indicate the survival curves of patients in high mesenchymal transition activity and low mesenchymal transition activity, respectively.

b. Kaplan–Meier survival analysis of overall survival of each cancer types based on the EMTCG score in TCGA cohort. The red and blue curves indicate the survival curves of patients in high mesenchymal transition activity and low mesenchymal transition activity, respectively.

Supplementary Figure 4 Pairwise comparison of the mesenchymal transition activity in TCGA cohort across 16 cancer types between tumor and non-tumor tissue.

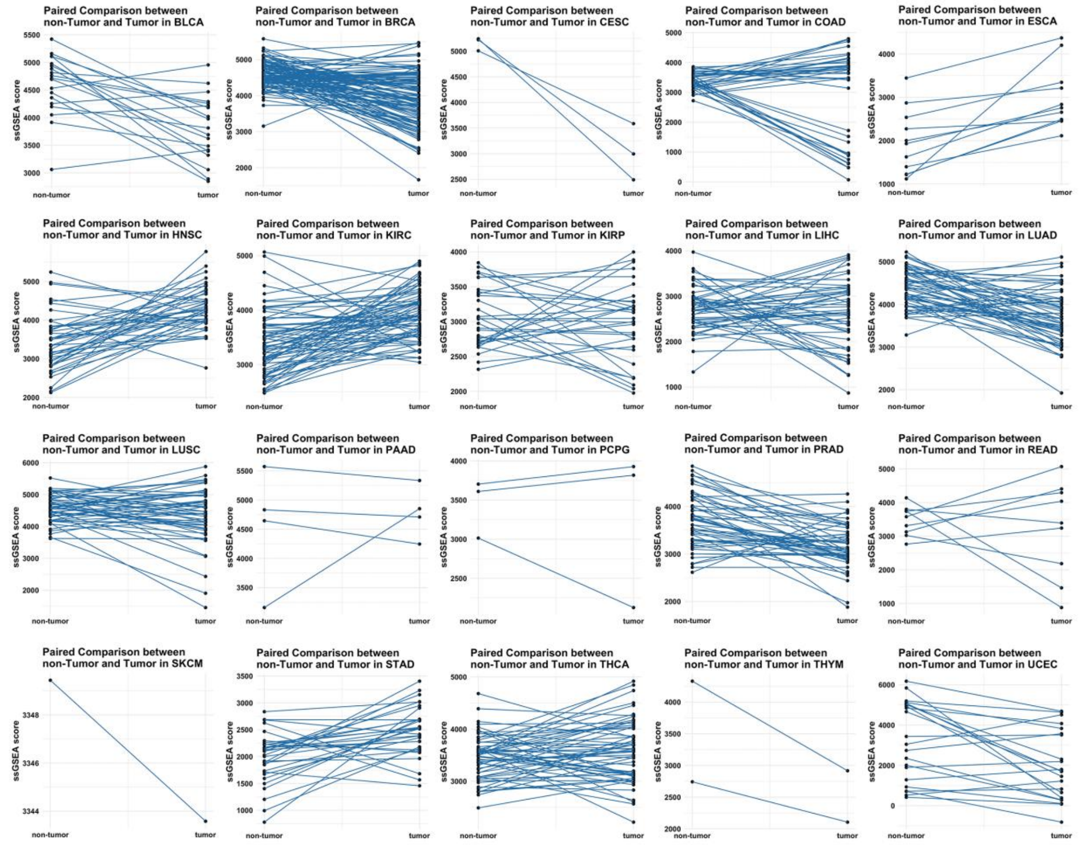

Mesenchymal transition activity was compared in 16 cancer types in TCGA cohort with paired normal tissue and tumor tissue. Plots in the left of the graph indicated mesenchymal transition activity of paired normal tissue while plots in the right of the graph indicated mesenchymal transition activity of paired tumor tissue. These graphs indicated although the mesenchymal transition activity was enhanced in most tumor compartments, the mesenchymal transition activity was inhibited compared to paired normal samples in some tumor compartments.

Supplementary Figure 5 Consensus Cluster of 9415 epithelial tumor samples in TCGA cohort using EMTCGs to classify epithelial tumors into for clusters according to EMT status.

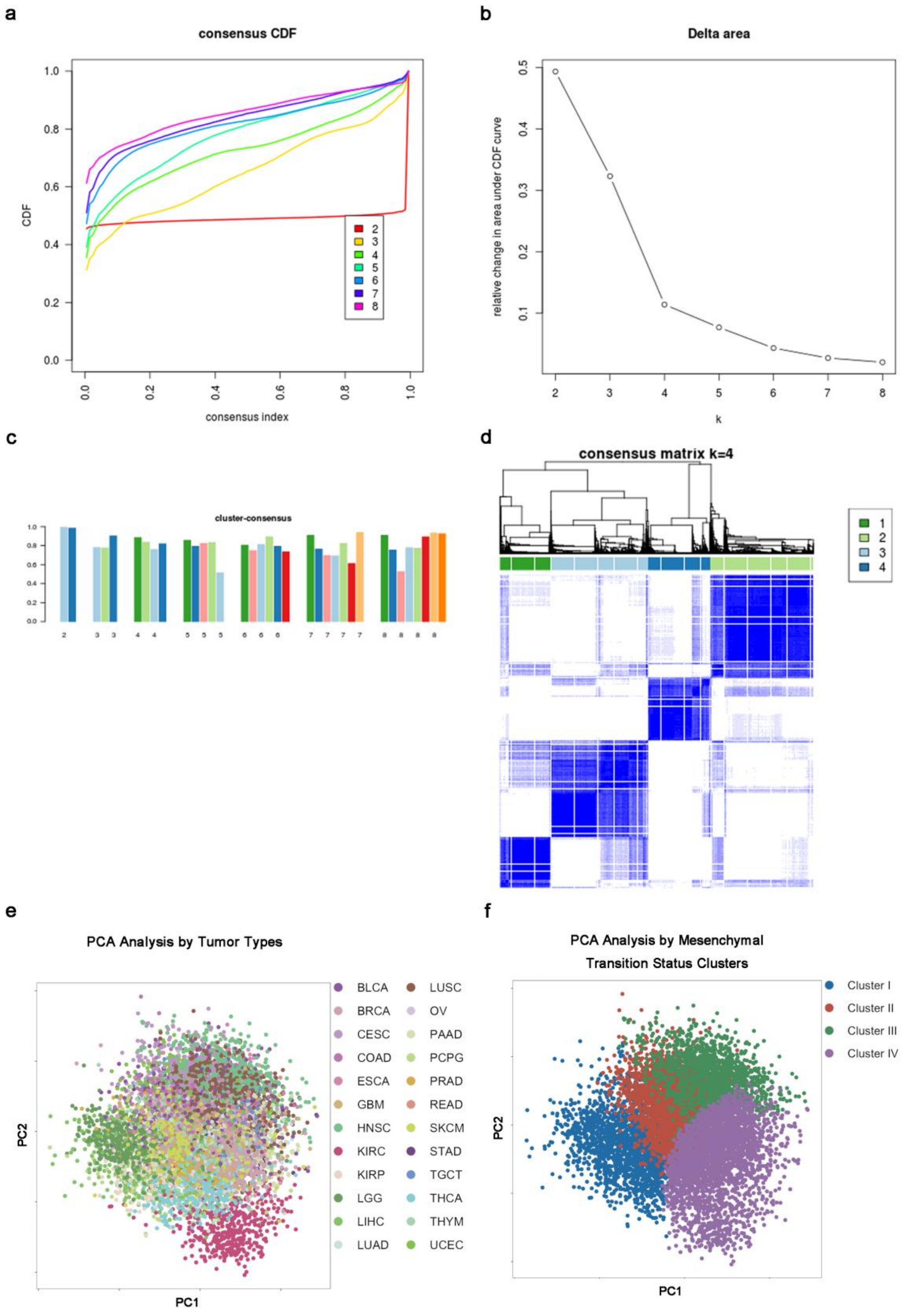

a. The cumulative distribution function (CDF) curves in consensus cluster analysis. CDF curves of consensus scores by different subtype number (k = 2, 3, 4, 5, 6, 7, 8) were represented.

b. Relative change of areas under CDF curves. The x-axis indicates number of subtypes. The y-axis represents the increased proportion of areas under CDF curve.

c. Cluster-consensus plot in consensus cluster analysis.

d. Consensus clustering of pan-cancer samples with k = 4.
e. Principal component analysis revealed that the distribution of 9415 samples from 24 tumor types in TCGA cohort were in chaos before cluster.
f. Principal component analysis for 35 EMTCGs to distinguish 24 different tumor types in TCGA cohort. PCA results exhibited that four EMT subtypes could be separated in different directions based on 35 EMTCGs.

Supplementary Figure 6 Two EMT^{high} subtypes have different survival status.

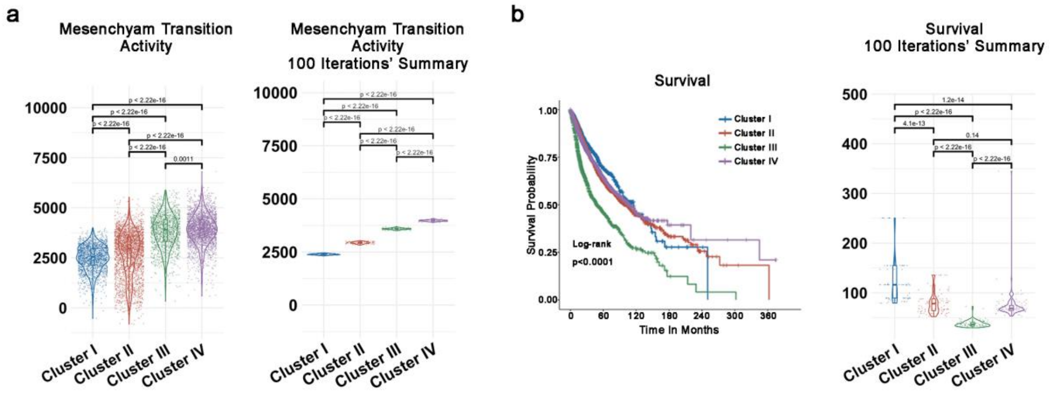

a. Comparison of mesenchymal transition activity among four clusters. The mesenchymal transition activity was significantly upregulated in Cluster III and IV; the Student's t test was used to analyze statistical significance; left panel: Comparison in pancancer scale; right panel: Summary of 100 iterations, each point represents mean ssGSEA score in each iteration; Cluster I, n=1864; Cluster II, n=3094; Cluster III, n=1532; Cluster IV, n=2925.

b. Kaplan-Meier curve of overall survival of patients with different EMT subtypes for TCGA datasets. Cluster III was statistically associated with unfavorable survival outcome; left panel: Comparison in pancancer scale; right panel: Summary of 100 iterations, each point represents mean survival in each iteration; Cluster I, n=1864; Cluster II, n=3094; Cluster III, n=1532; Cluster IV, n=2925.

Supplementary Figure 7 Tumor Microenvironment Analysis

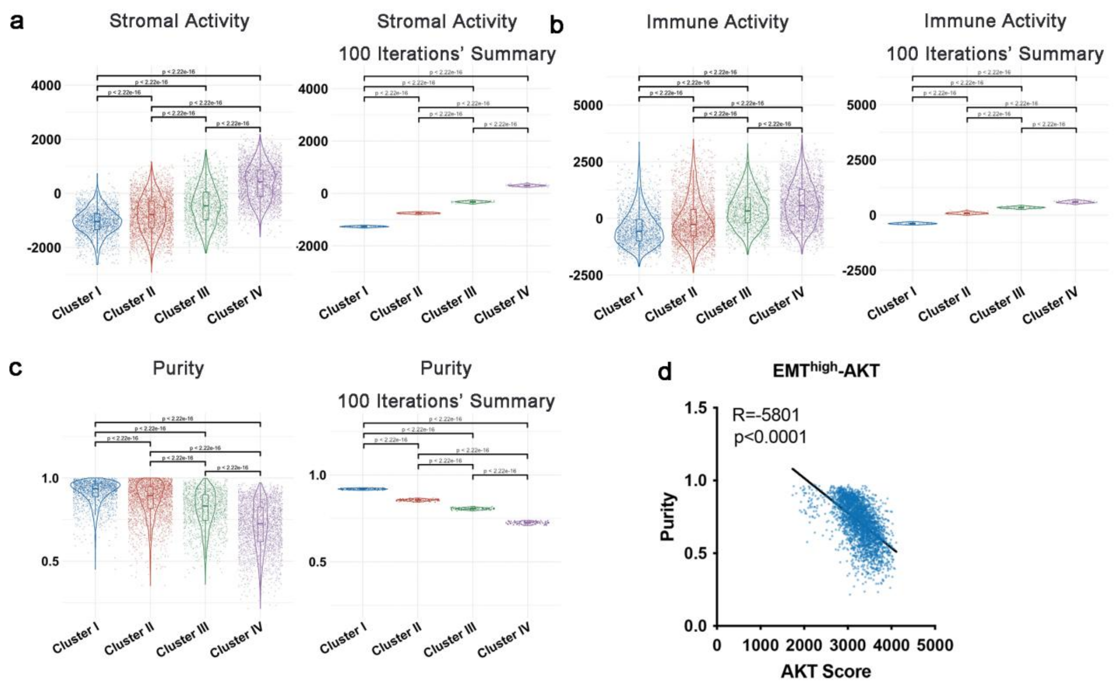

a. Comparison of Stromal Score among four EMT subtypes in TCGA cohort. EMT^{high}-AKT subtype had the highest Stromal Score among the four EMT subtypes in 100 iteration repeats; Student's t test was used to analyze statistical significance between 2 groups.

b. Comparison of Immune Score among four EMT subtypes in TCGA cohort. EMT^{high}-AKT subtype had the highest Immune score among the four EMT subtypes in 100 iteration repeats; Student's t test was used to analyze statistical significance between 2 groups.

c. Comparison of tumor purity among four EMT subtypes in TCGA cohort. EMT^{high}-AKT subtype had the lowest tumor purity among the four EMT subtypes in 100 iteration repeats; Student's t test was used to analyze statistical significance between 2 groups.

d. AKT pathway score was negatively correlated with tumor purity in EMT^{high}-AKT subtype in TCGA cohort.

For a-c, left panel: Comparison in pancancer scale; right panel: Summary of 100 iterations, each point represents mean score in each iteration; ; Cluster I, n=1864; Cluster II, n=3094; Cluster III, n=1532; Cluster IV, n=2925.

Supplementary Figure 8 Multi-scale comparison between EMT^{high}-NOS subtype and EMT^{high}-AKT subtype in TCGA cohort

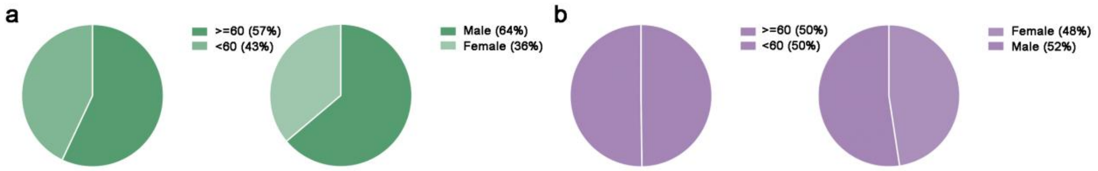

a. Left panel: Age composition of EMT^{high}-NOS subtype; Right panel: Gender composition of EMT^{high}-NOS subtype.

b. Left panel: Age composition of EMT^{high}-AKT subtype; Right panel: Gender composition of EMT^{high}-AKT subtype.

Supplementary Figure 9 Identification of driving pathways

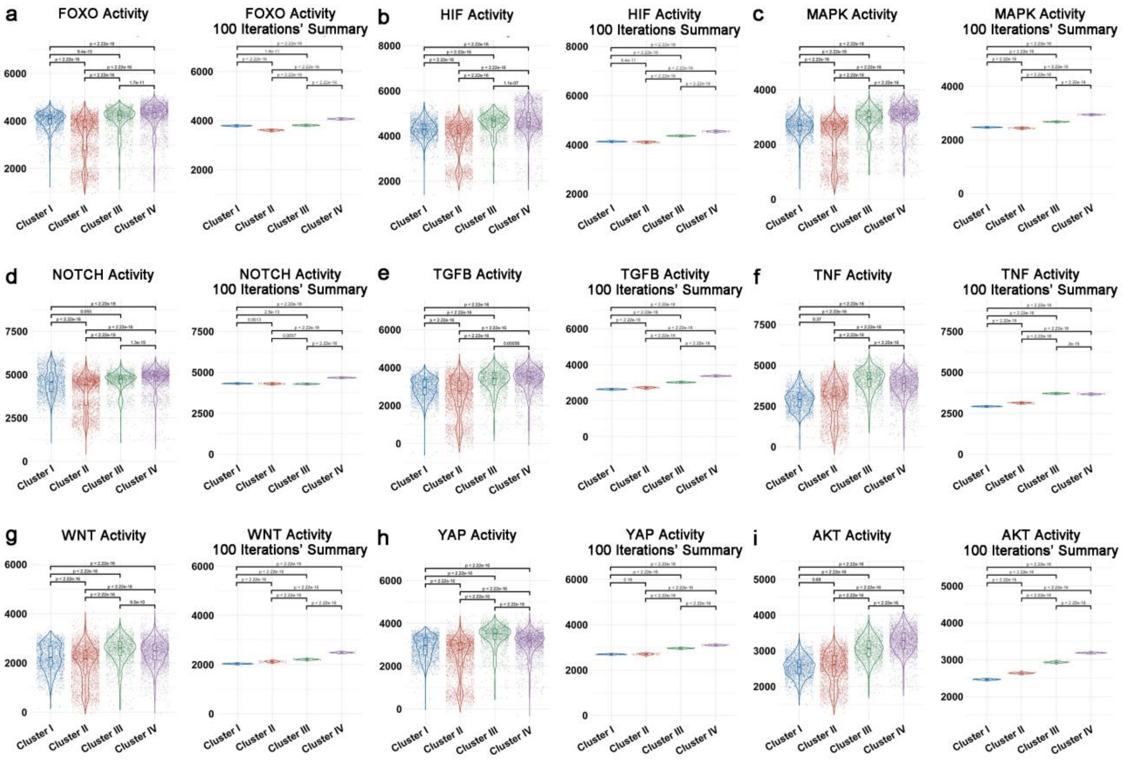

a. Comparison of FOXO pathway activity among four EMT subtypes in TCGA cohort.

b. Comparison of HIF pathway activity among four EMT subtypes in TCGA cohort.

c. Comparison of MAPK pathway activity among four EMT subtypes in TCGA cohort.

d. Comparison of NOTCH pathway activity among four EMT subtypes in TCGA cohort.

e. Comparison of TGFB pathway activity among four EMT subtypes in TCGA cohort.

f. Comparison of TNF pathway activity among four EMT subtypes in TCGA cohort.

g. Comparison of WNT pathway activity among four EMT subtypes in TCGA cohort.

h. Comparison of YAP pathway activity among four EMT subtypes in TCGA cohort.

i. Comparison of AKT pathway activity among four EMT subtypes in TCGA cohort.

For all graphs in this figure, left panel: Comparison in pancancer scale; right panel: Summary of 100 iterations, each point represents mean ssGSEA score in each iteration; Student's t test was used to analyze statistical significance between 2 groups; Cluster I, n=1864; Cluster II, n=3094; Cluster III, n=1532; Cluster IV, n=2925.

Supplementary Figure 10 AKT pathway is overactivated in EMT^{high}-AKT subtype in cancer cell lines

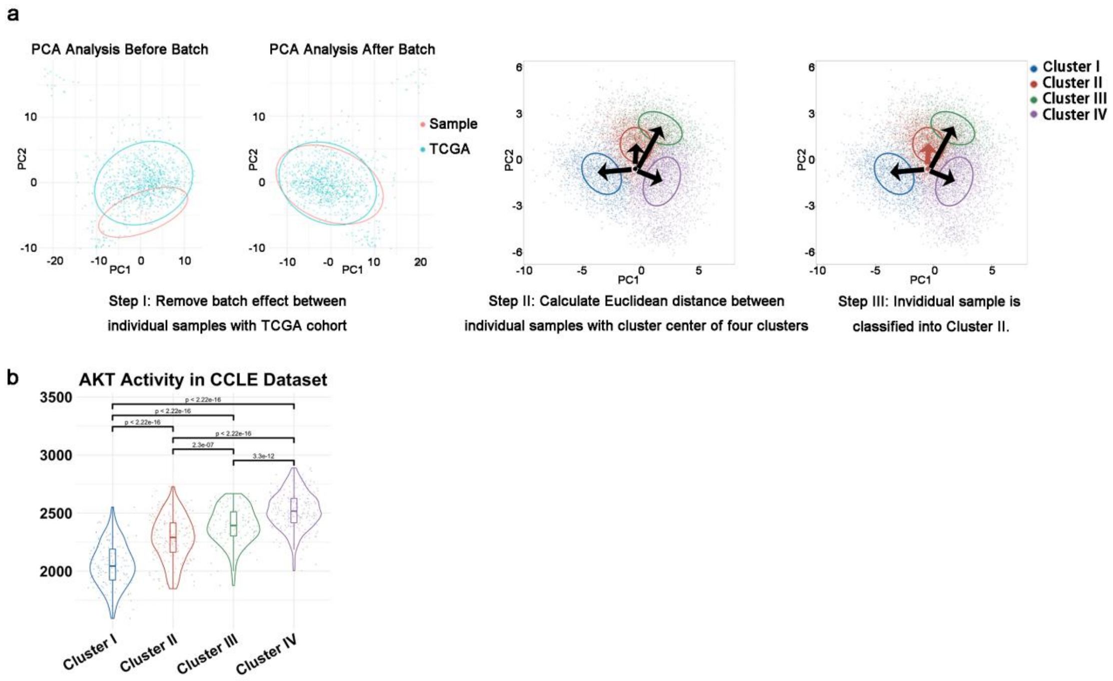

a. Schematic plot to reclassify individual samples into 35 EMTCGs-based EMT subtypes.

b. Comparison of AKT pathway activity score among different EMT clusters in CCLE dataset; EMT^{high}-AKT subtype had the highest AKT pathway activity among the four EMT subtypes; Student's t test was used to analyze statistical significance between 2 groups; Cluster I, n=1864; Cluster II, n=3094; Cluster III, n=1532; Cluster IV, n=2925.

Supplementary Figure 11 Immunohistochemistry validation

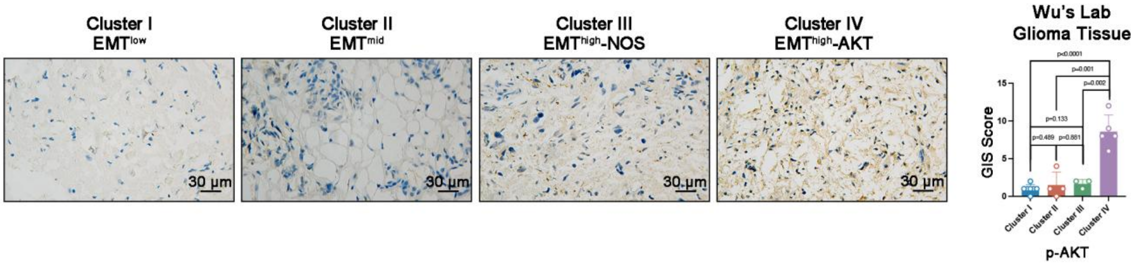

Immunohistochemistry indicates phosphorylation level of AKT is significantly upregulated in EMT^{high}-AKT subtype; Scale bar: 30 μ m.

Supplementary Figure 12 Only EMT^{high}-AKT but not EMT^{high}-NOS subtype was resistant to targeting drugs

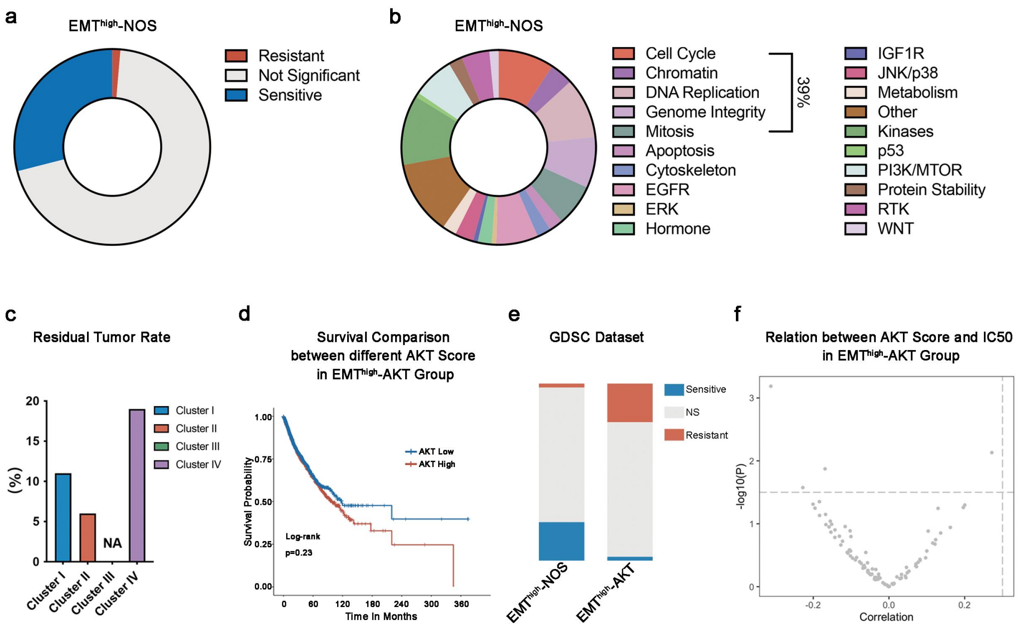

a. Among 445 drugs chemotherapeutic drugs, EMT^{high}-NOS subtype was sensitive to 129 drugs (29%) and resistant to 6 (1%) drugs.

b. The drug classification of 129 sensitive drugs in EMT^{high}-NOS subtype.

c. The residual tumor rates in four EMT subtypes.

d. The overall survival didn't shown significance between low AKT pathway activity group and high AKT pathway activity group in EMT^{high}-AKT subtype.

e. Compared to EMT^{high}-NOS subtype, 97 chemotherapeutic drugs were resistant in EMT^{high}-AKT subtype.

f. The IC50s of 97 resistant chemotherapeutic drugs in EMT^{high}-AKT subtype were not correlated with AKT pathway activity.

Supplementary Figure 13 EMT^{high}-AKT subtype tumors exemplify a T cell-dysfunction immune-suppressive phenotype caused by M2-TAMs

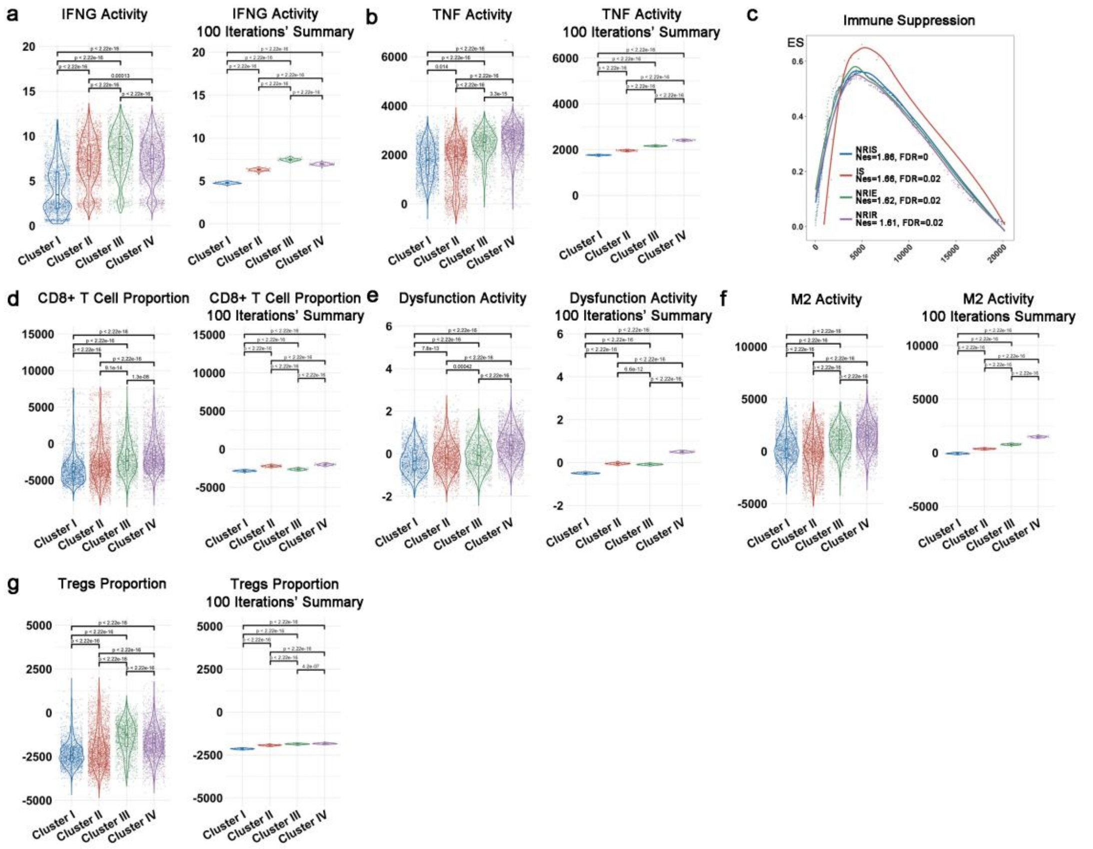

a. Comparison of IFNG activity between the four EMT subtypes in TCGA cohort. EMT^{high}-NOS subtype had the highest IFNG activity among the four EMT clusters in 100 iteration times; Cluster I, n=1842; Cluster II, n=2836; Cluster III, n=1506; Cluster IV, n=2873.

b. Comparison of TNFA activity between the four EMT subtypes in TCGA cohort. EMT^{high}-AKT subtype had the highest TNFA activity among the four EMT clusters in 100 iteration times.

c. GSEA analyses displayed immunosuppressive gene signatures enriched in EMT^{high}-AKT subtype. FDR < 0.05 was considered as statistically significant.

d. Comparison of CD8+ T cell proportion between the four EMT subtypes in TCGA cohort; EMT^{high}-AKT subtype had similar CD8+ T cell infiltration level with other three EMT clusters in 97 iteration times.

e. Comparison of T-cell dysfunction score between the four EMT subtypes in TCGA cohort; EMT^{high}-AKT subtype had the highest T-cell dysfunction score among the four EMT clusters in 100 iteration times.

f. Comparison of M2-TAMs proportion between the four EMT subtypes in TCGA cohort; EMT^{high}-AKT subtype had the highest M2-TAMs fraction among the four EMT clusters in 100 iteration times.

g. Comparison of Tregs proportion between the four EMT subtypes in TCGA cohort. EMT^{high}-AKT subtype had similar Tregs infiltration level with EMT^{high}-AKT subtype in 98 iteration times.

For a, b and d-g in this figure, Student's t test was used to analyze statistical significance between 2 groups; left panel: Comparison in pancancer scale; right panel: Summary of 100 iterations, each point represents mean score in each iteration.

For b and d-g in this figure, Cluster I, n=1864; Cluster II, n=3094; Cluster III, n=1532; Cluster IV, n=2925.

Supplementary Figure 14 AKT pathway is important for mediating mesenchymal transition and immunosuppression only in EMT^{high}-AKT subtype

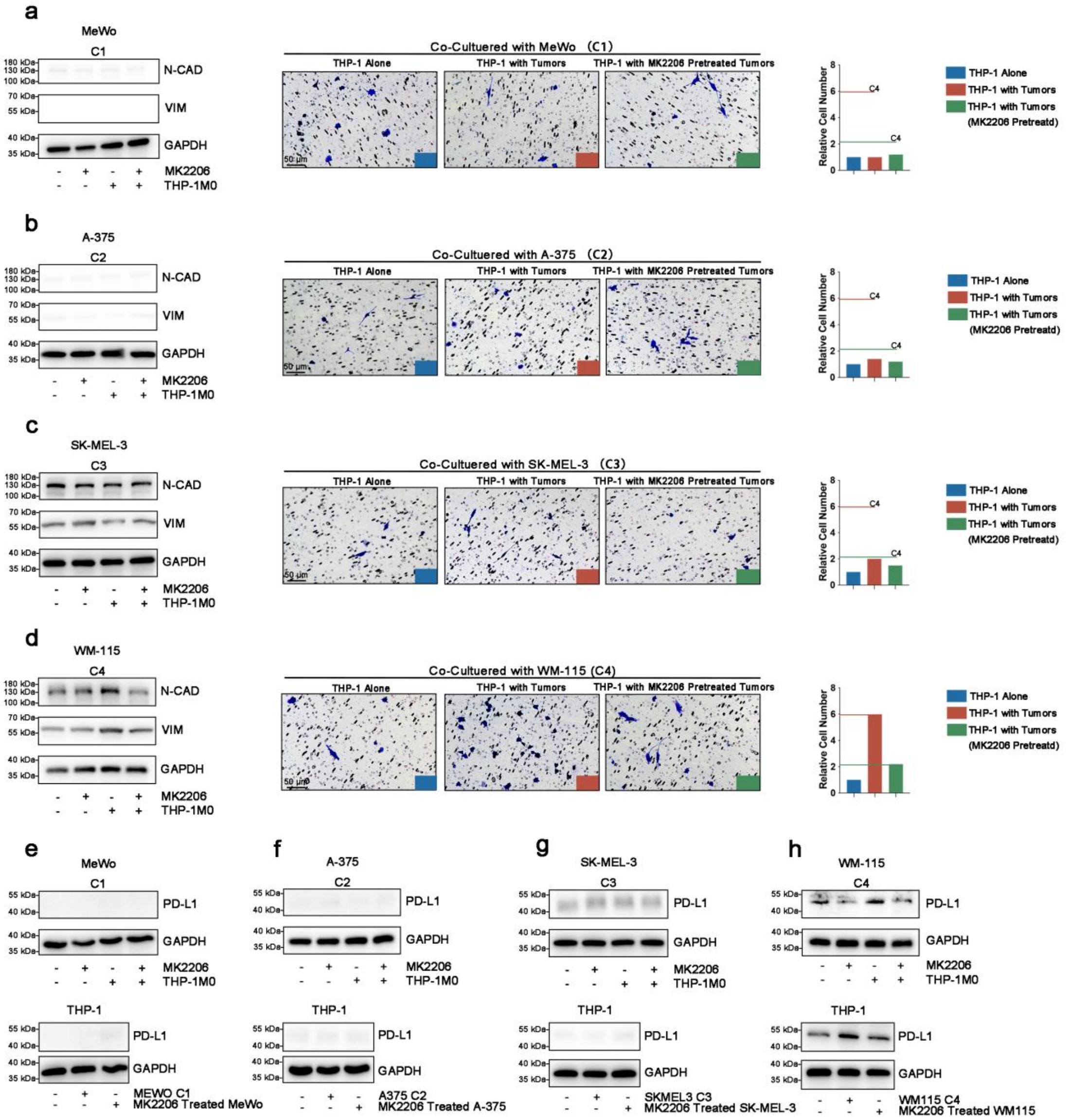

a. Left panel: Immunoblotting analysis of N-Cadherin and Vimentin indicated that macrophage could not promote mesenchymal transition of MeWo. Right panel: Transwell analysis indicated MeWo could not enhance infiltration ability of macrophages.

b. Left panel: Immunoblotting analysis of N-Cadherin and Vimentin indicated that macrophage could not promote mesenchymal transition of A-375. Right panel: Transwell analysis indicated A-375 enhanced infiltration ability of macrophages mildly and could not be inhibited by AKT inhibition.

c. Left panel: Immunoblotting analysis of N-Cadherin and Vimentin indicated that macrophage could not enhance mesenchymal transition of SK-MEL-3. Right panel: Transwell analysis indicated SK-MEL-3 enhanced infiltration ability of macrophages mildly.

d. Left panel: Immunoblotting analysis of N-Cadherin and Vimentin indicated that macrophage could enhance mesenchymal transition of WM-115 which could be inhibited by AKT inhibition. Right panel: Transwell analysis indicated WM-115 enhanced infiltration ability of macrophages dramatically and

could be inhibited by AKT inhibition.

e. PD-L1 expression did not upregulate in co-cultured MeWo and macrophages.

f. PD-L1 expression did not upregulate in co-cultured A-375 and macrophages.

g. PD-L1 expression did not upregulate in co-cultured SK-MEL-3 and macrophages.

h. PD-L1 expression upregulated in co-cultured WM-115 and macrophages which could be inhibited by AKT inhibition.

For all transwell images in this figure, scale bar = 50 μ m.

Supplementary Figure 15 AKT pathway inhibition could block M2 transformation of macrophages in tumor tissue

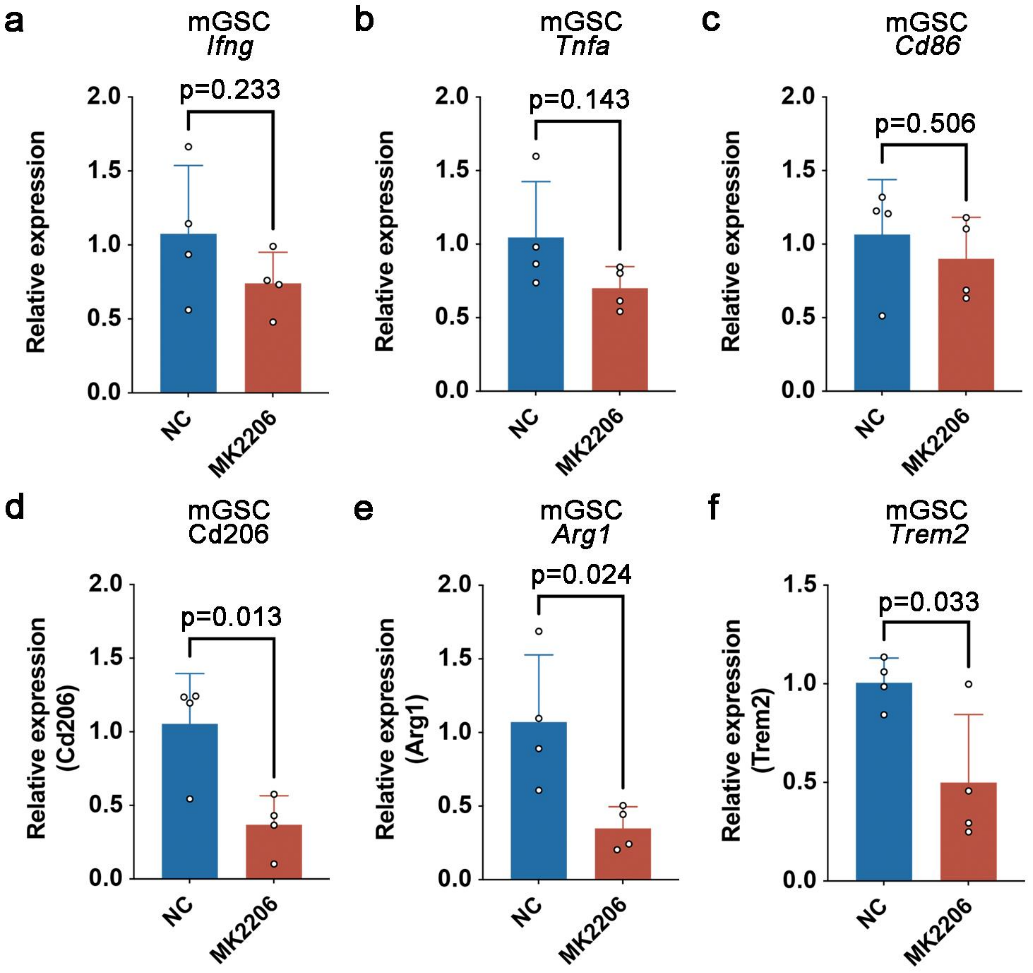

a. Ifng expression didn't alter in mGSC xenograft model after MK2206 administration.

b. Tnfa expression didn't alter in mGSC xenograft model after MK2206 administration.

c. Cd86 expression didn't alter in mGSC xenograft model after MK2206 administration.

d. Cd206 expression downregulated in mGSC xenograft model after MK2206 administration.

e. Arg1 expression downregulated in mGSC xenograft model after MK2206 administration.

f. Trem2 expression downregulated in mGSC xenograft model after MK2206 administration.

n = 4 biological replicates; Error bars indicate SD; Student's t test was used to analyze statistical significance between 2 groups.

Supplementary Figure 16 Immune cell distribution between NC group and MK2206 group in mGSC xenograft model

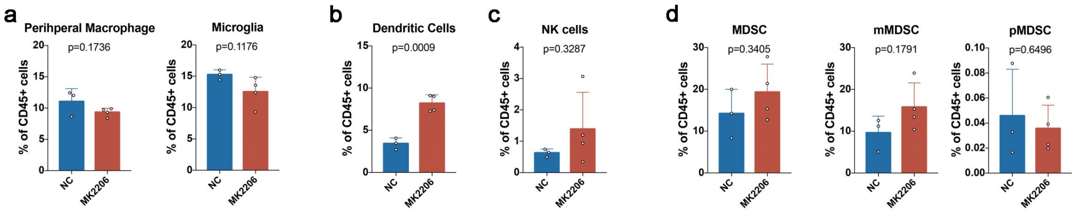

a. Peripheral macrophage or microglia infiltration did not alter between NC group and MK2206 group in mGSC xenograft model.

b. More dendritic cells infiltrated into MK2206 group compared to NC group in mGSC xenograft model.

c. NK cell content didn't alter between NC group and MK2206 group in mGSC xenograft model.

d. MDSC cell content didn't alter between NC group and MK2206 group in mGSC xenograft model.

Error bars indicate SD; the Student's t test was used to analyze statistical significance between 2 groups;

In NC group, n=3 biological replicates; In MK2206 group, n=4 biological replicates.

Supplementary Figure 17 Drug administration strategy of ICBs plus MK2206 treatment of in vivo test

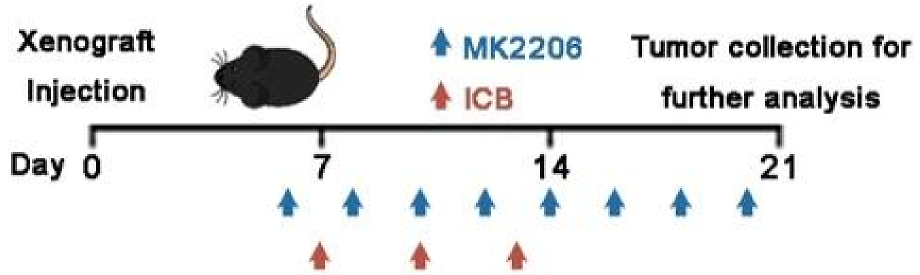

MK2206 was orally administered on days 6, 8, 10, 12, 14, 16, 18, and 20 at a dose of 150 μg/g body weight. Anti-ICBs were intraperitoneally injected on days 7, 10, and 13 at a dose of 10 μg/g body weight. ICBs include anti-PD-L1, anti-PD-1 or anti-CTLA-4.

Supplementary Figure 18 MK2206 didn't cause damage to liver and kidney in xenograft model

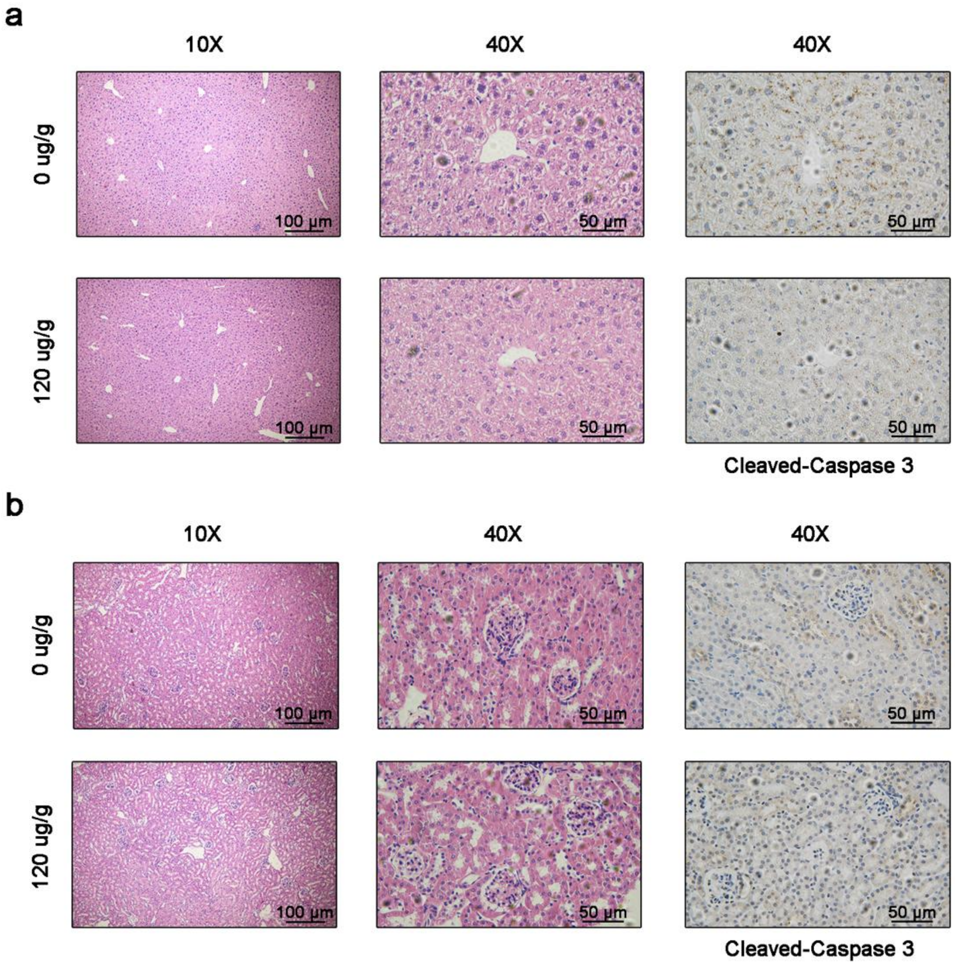

a. Left panel: MK2206 didn't cause structure damage in liver; right panel: MK2206 didn't cause apoptosis of liver cells.

b. Left panel: MK2206 didn't cause structure damage in kidney; right panel: MK2206 didn't cause apoptosis of kidney cells.

For 10X images in this figure, scale bar = 100 μ m; for 40X images in this figure, scale bar = 50 μ m.

Supplementary Figure 19 Schema of a novel EMT classification system

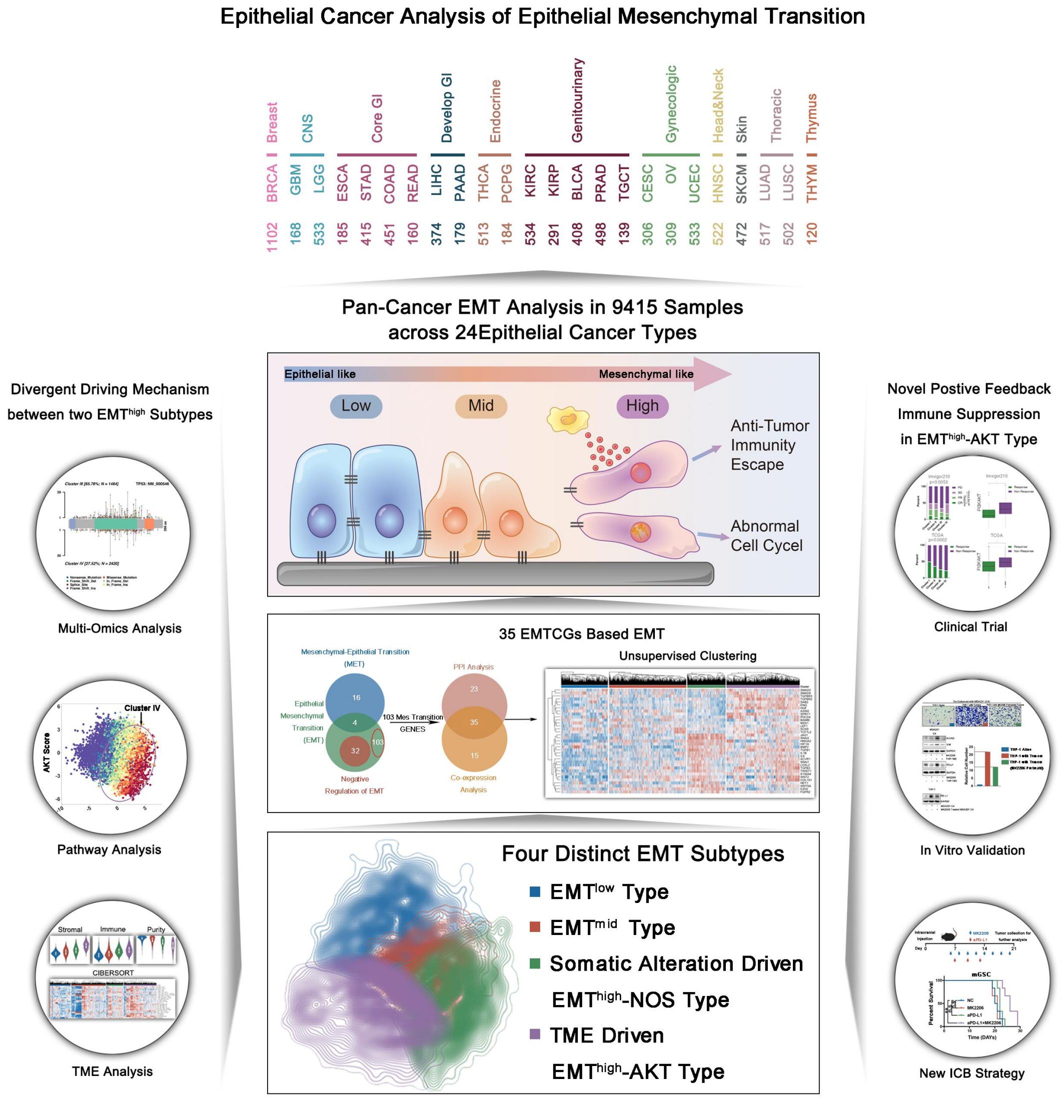

9415 samples from 24 cancer types in TCGA cohort could be divided into four EMT subtypes using 35 EMTCG genes with distinct driving mechanisms and should be treated with different strategies.

Supplementary Figure 20 Flowchart for using the Tumor MT resource.

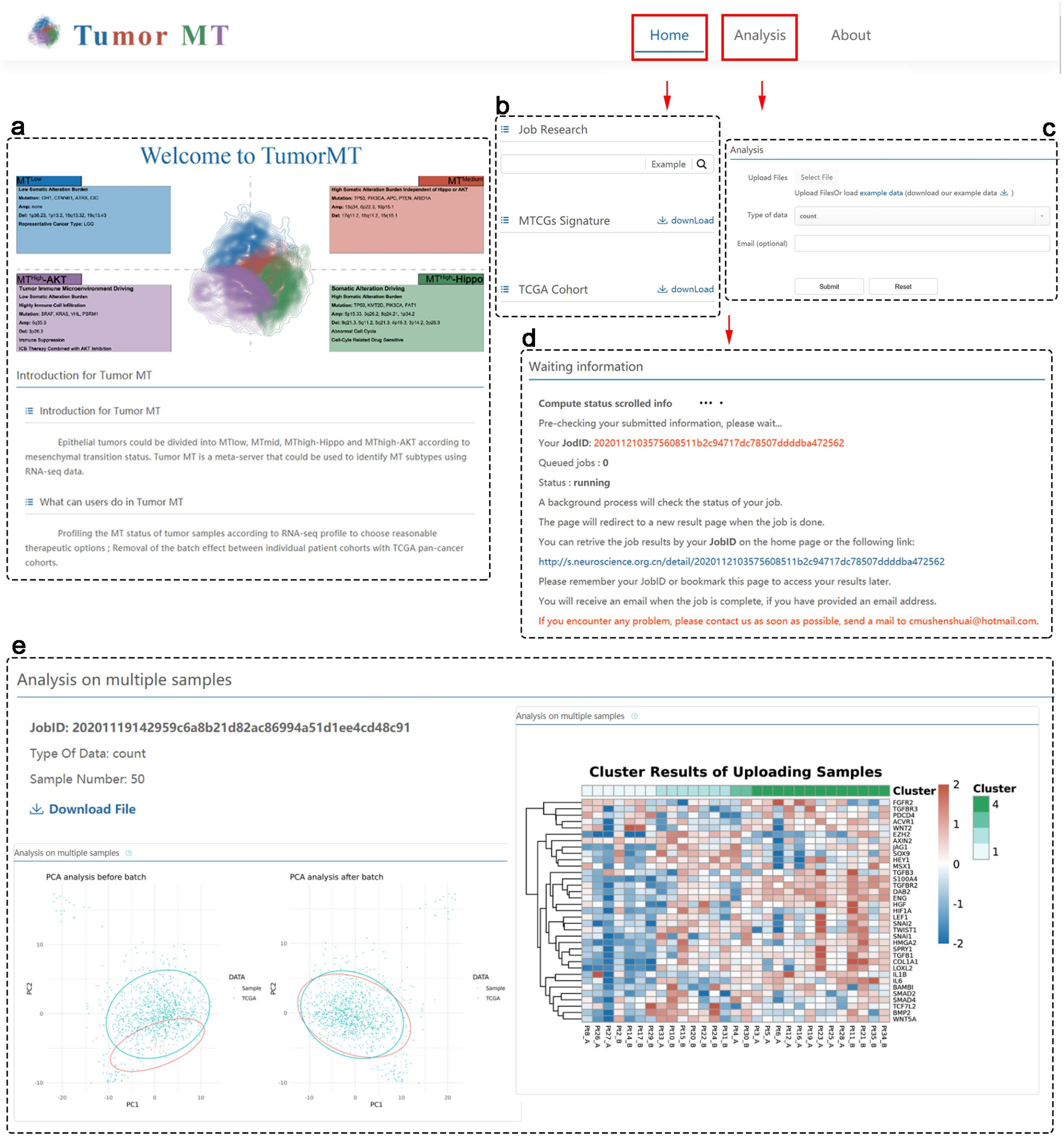

a. Home page of Tumor MT.

b. Resources download part of Tumor MT.

c. Analysis part of Tumor MT.

d. Page waiting during background analysis.

e. Result part of Tumor MT.

Supplementary Figure 21 Validation in CGGA GBM dataset

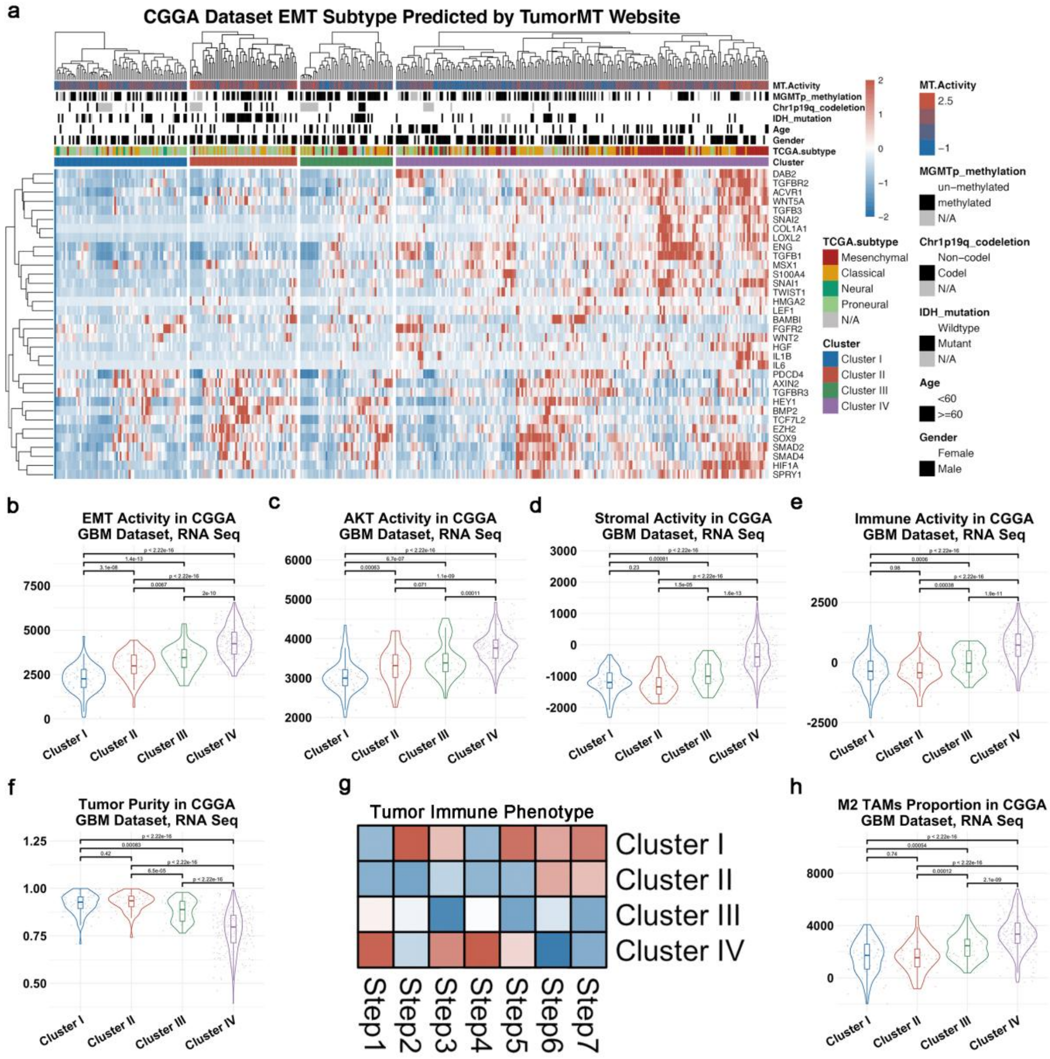

a. CGGA GBM samples could be classified into four EMT subtypes.

b. Comparison of EMT activity among four EMT subtypes in CGGA GBM cohort and EMT^{high}-AKT subtype had the highest AKT pathway activity among the four EMT subtypes.

c. Comparison of AKT pathway activity among four EMT subtypes in CGGA GBM cohort and EMT^{high}-AKT subtype had the highest AKT pathway activity among the four EMT subtypes.

d. Comparison of Stromal Score among four EMT subtypes in CGGA GBM cohort and EMT^{high}-AKT subtype had the highest Stromal Score among the four EMT subtypes.

e. Comparison of Immune Score among four EMT subtypes in CGGA GBM cohort and EMT^{high}-AKT subtype had the highest Immune Score among the four EMT subtypes.

f. Comparison of tumor purity among four EMT clusters in CGGA GBM cohort and EMT^{high}-AKT subtype had the lowest tumor purity among the four EMT subtypes.

g. Tracking Tumor Immunophenotype of four EMT subtypes in CGGA GBM cohort.

h. Comparison of M2-TAMs fraction among four EMT subtypes in CGGA GBM cohort and EMT^{high}-AKT subtype had the highest M2-TAMs fraction among the four EMT subtypes.

For b-f and h, Student's t test was used to analyze statistical significance between 2 groups; Cluster I, n=73; Cluster II, n=59; Cluster III, n=51; Cluster IV, n=205.

Supplementary Figure 22 Uncropped and unedited blot images in this work

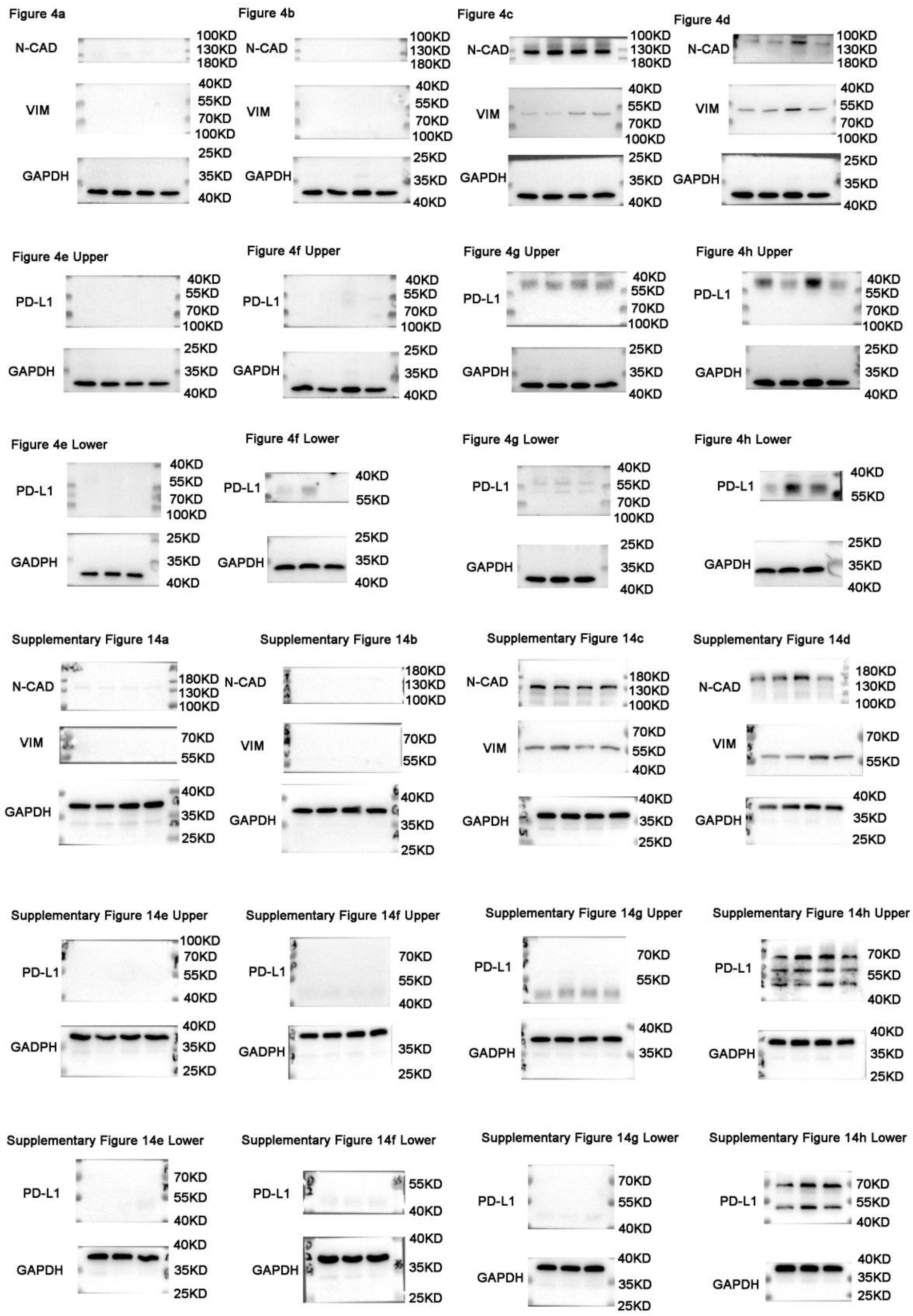

Supplement: Supplementary file 2 — Supplementary Information [file 42003_2023_5667_MOESM2_ESM.pdf]
