# Supplementary material for: Tumor microenvironment remodeling plus immunotherapy could be used in mesenchymal-like tumor with high tumor residual and drug resistant rate
Source: Commun Biol. 2023 Dec 18;6:1281. doi: 10.1038/s42003-023-05667-4 (PMC10728080; doi:10.1038/s42003-023-05667-4)
Supplement: Supplementary file 3 — Description of Additional Supplementary Files [file 42003_2023_5667_MOESM3_ESM.pdf]

Description of Additional Supplementary Files

File name: Supplementary Data 1
Description: Mesenchymal Transition Gene Set

File name: Supplementary Data 2
Description: Protein-protein Interaction

File name: Supplementary Data 3
Description: Mutation Regulation Mechanism of EMTCGs

File name: Supplementary Data 4
Description: CNV Regulation Mechanism of EMTCGs

File name: Supplementary Data 5
Description: Methylation Mechanism of EMTCGs

File name: Supplementary Data 6
Description: Mesenchymal Transition Activity Score

File name: Supplementary Data 7
Description: COX Analysis of Mesenchymal transition activity

File name: Supplementary Data 8
Description: Coefficient of Variation of Mesenchymal transition activity

File name: Supplementary Data 9
Description: Driver Mutation among Four EMT Clusters

File name: Supplementary Data 10
Description: Driver CNV among Four EMT Clusters

File name: Supplementary Data 11
Description: Normalization of Sample Number in each variant

File name: Supplementary Data 12
Description: KEGG Analysis of EMTCGs

File name: Supplementary Data 13
Description: CCLE EMT Cluster Result

File name: Supplementary Data 14
Description: DEG between EMT-High-NOS and EMT-High-AKT subtype

File name: Supplementary Data 15
Description: GO Analysis of EMT-High-NOS and EMT-High-AKT subtype

File name: Supplementary Data 16
Description: CyTOF Panel

File name: Supplementary Data 17
Description: Sample Number Summary

File name: Supplementary Data 18
Description: Reagents, Antibodies, Softwares and Equipments

File name: Supplementary Data 19
Description: Numerical source data for graphs and charts
