# Supplementary material for: Tumor microenvironment remodeling plus immunotherapy could be used in mesenchymal-like tumor with high tumor residual and drug resistant rate
Source: Commun Biol. 2023 Dec 18;6:1281. doi: 10.1038/s42003-023-05667-4 (PMC10728080; doi:10.1038/s42003-023-05667-4)
Supplement: Supplementary file 5 — Reporting Summary [file 42003_2023_5667_MOESM5_ESM.pdf]

nature portfolio

Corresponding author(s): Anhua Wu, Wen Cheng and Peng Cheng

Last updated by author(s): Nov 1, 2023

Reporting Summary

Nature Portfolio wishes to improve the reproducibility of the work that we publish. This form provides structure for consistency and transparency in reporting. For further information on Nature Portfolio policies, see our Editorial Policies and the Editorial Policy Checklist.

Statistics

For all statistical analyses, confirm that the following items are present in the figure legend, table legend, main text, or Methods section.

n/a	Confirmed	
☐	☒	The exact sample size (*n*) for each experimental group/condition, given as a discrete number and unit of measurement
☐	☒	A statement on whether measurements were taken from distinct samples or whether the same sample was measured repeatedly
☐	☒	The statistical test(s) used AND whether they are one- or two-sided<br>*Only common tests should be described solely by name; describe more complex techniques in the Methods section.*
☐	☒	A description of all covariates tested
☐	☒	A description of any assumptions or corrections, such as tests of normality and adjustment for multiple comparisons
☐	☒	A full description of the statistical parameters including central tendency (e.g. means) or other basic estimates (e.g. regression coefficient) AND variation (e.g. standard deviation) or associated estimates of uncertainty (e.g. confidence intervals)
☐	☒	For null hypothesis testing, the test statistic (e.g. *F*, *t*, *r*) with confidence intervals, effect sizes, degrees of freedom and *P* value noted<br>*Give P values as exact values whenever suitable.*
☒	☐	For Bayesian analysis, information on the choice of priors and Markov chain Monte Carlo settings
☒	☐	For hierarchical and complex designs, identification of the appropriate level for tests and full reporting of outcomes
☒	☐	Estimates of effect sizes (e.g. Cohen's *d*, Pearson's *r*), indicating how they were calculated

Our web collection on statistics for biologists contains articles on many of the points above.

Software and code

Policy information about availability of computer code

Data collection	R version 3.5.1 (http://research-pub.gene.com/IMvigor210CoreBiologies/packageVersions/)
Data analysis	Prism 7; R version 3.5.1 (GSVA R package, survival R package, ConsensusClusterPlus R package, ggplot2 R package, survminer R package, maftools R package, estimate R package, affy R package, limma R package, sva R package), Python 3.9 (sklearn package [KNN algorithm, LDA algorithm, LR algorithm], pandas package, scipy package, numpy package, matplotlib package), Perl 5.30.2 (quartile_norm.pl)

For manuscripts utilizing custom algorithms or software that are central to the research but not yet described in published literature, software must be made available to editors and reviewers. We strongly encourage code deposition in a community repository (e.g. GitHub). See the Nature Portfolio guidelines for submitting code & software for further information.

Data

Policy information about availability of data

All manuscripts must include a data availability statement. This statement should provide the following information, where applicable:

- Accession codes, unique identifiers, or web links for publicly available datasets
- A description of any restrictions on data availability
- For clinical datasets or third party data, please ensure that the statement adheres to our policy

TCGA multi-omics data (clinical information ‘2018-09-13 version’, copy number variation profile ‘2016-08-16 version’, gene mutation profile ‘2016-12-29 version’, DNA methylation profile ‘Methylation450K 2016-12-29 version’, transcriptomic expression profile ‘2016-12-29 version, RNA seq’, and reverse-phase protein array

profile '2016-08-16 version') were downloaded from the Xena Website (https://xenabrowser.net/). The cancer cell line transcriptomic expression profile '20180929 version, RNA seq' was downloaded from Cancer Cell Line Encyclopedia (CCLE) Website (https://portals.broadinstitute.org/ccle/). Colorectal cancer datasets (KFSYSCC cohort, FRENCH cohort, GSE2109 cohort, GSE37892 cohort, GSE35896 cohort, GSE23878 cohort, GSE20916 cohort, GSE17536 cohort, and GSE13067 cohort; Transcriptome) were downloaded as the authors indicated. Breast cancer dataset (FUSCCTNBC cohort; Transcriptome) was downloaded as the authors indicated. Gastric cancer datasets (ACRG cohort, KUCM cohort, KUGH cohort, MDACC cohort and SMC cohort; Transcriptome) were downloaded as the author indicated. IMvigor210CoreBiologies data (RNA seq) were downloaded using the R package (version 1.0.0) provided by the following website (http://research-pub.gene.com/IMvigor210CoreBiologies/packageVersions/). GSE78220 (RNA seq) data was downloaded from GEO website; Anti-CTLA4 clinical trial data (RNA seq) was acquired from dbGaP: phs000452 (https://github.com/vanallenlab/VanAllen_CTLA4_Science_RNASeq_TPM/commit/3d1793629716cc1fd8e7334ea3bf593a20e6fe07) and SRA: SRP067586 cohort. The CGGA cohort data could be obtained from the CGGA database (http://www.cgga.org.cn). A total of 292 multiregional glioma tissue samples from 71 patients were acquired from our institution with the assistance of neuronavigation between October 10, 2020 and August 18, 2022.

Human research participants

Policy information about studies involving human research participants and Sex and Gender in Research.

Reporting on sex and gender	We have uploaded the sex and gender information of the participants with raw and processed RNA-seq data.
Population characteristics	Glioma
Recruitment	N/A
Ethics oversight	The experimental protocol was approved by the ethics committee of The First Hospital of China Medical University.

Note that full information on the approval of the study protocol must also be provided in the manuscript.

Field-specific reporting

Please select the one below that is the best fit for your research. If you are not sure, read the appropriate sections before making your selection.

☒ Life sciences ☐ Behavioural & social sciences ☐ Ecological, evolutionary & environmental sciences

For a reference copy of the document with all sections, see nature.com/documents/nr-reporting-summary-flat.pdf

Life sciences study design

All studies must disclose on these points even when the disclosure is negative.

Sample size	For each cancer type in TCGA analysis, the sample number must be larger than 50.
Data exclusions	In TCGA analysis, if the sample number was smaller than 50, then this type of cancer was excluded. For CyTOF analysis, by preliminary analysis, one sample in NC group was obviously distinct from others and was removed in further analysis as indicated by test company.
Replication	For CyTOF Analysis, 3 replicates were analyzed in NC group, 4 replicates were analyzed in MK2206 group. For 4T1 animal experiments, 5 replicates were performed in each group. For B16F10/anti-PD-L1 animal experiments, 4 replicates were performed in each group. For B16F10/anti-PD-1 or B16F10/anti-CTLA-4 animal experiments, 5 replicates were performed in each group. For mGSC animal experiments, 6 replicates were performed in each group.
Randomization	For animal experiments, animals were randomly put into different groups before any operation was performed.
Blinding	Blinding was not possible, since the animal experiment operation and data collection was performed by the same group of people.

Reporting for specific materials, systems and methods

We require information from authors about some types of materials, experimental systems and methods used in many studies. Here, indicate whether each material, system or method listed is relevant to your study. If you are not sure if a list item applies to your research, read the appropriate section before selecting a response.

Materials & experimental systems

n/a	Involved in the study
☐	☒ Antibodies
☐	☒ Eukaryotic cell lines
☒	☐ Palaeontology and archaeology
☐	☒ Animals and other organisms
☐	☒ Clinical data
☒	☐ Dual use research of concern

Methods

n/a	Involved in the study
☒	☐ ChIP-seq
☒	☐ Flow cytometry
☒	☐ MRI-based neuroimaging

Antibodies

Antibodies used

GAPDH Monoclonal Antibody (Proteintech, 60004-1, 1E6D9), HRP-conjugated Affinipure Goat Anti-Mouse IgG(H+L) (Proteintech, SA00001-1), HRP-conjugated Affinipure Goat Anti-Rabbit IgG(H+L) (Proteintech, SA00001-2), Vimentin (D21H3) XP® Rabbit mAb (Cell Signaling Technology, 5741), Anti-PD-L1 Antibody (Abcam, ab213524), Phospho-AKT (Ser473) Monoclonal Antibody (Proteintech, 66444-1-Ig, 1C10B8), cleaved-Caspase 3 (Servicebio, GB11532),N-Cadherin Polyclonal Antibody (Proteintech, 22018-1-AP), InVivoMAb anti-mouse PD-L1 (BioXcell, BE0101), InVivoMAb anti-mouse CTLA-4 (BioXcell, BE0131), InVivoMAb anti-mouse PD-1 (BioXcell, BE0273).

Validation

All antibodies were used for the functions validated by the manufacture.

Eukaryotic cell lines

Policy information about cell lines and Sex and Gender in Research

Cell line source(s)

The human breast cancer cell lines MDA-MB-231 and MDA-MB-468 were purchased from the Chinese Academy of Sciences cell bank (Shanghai, China). The human breast cancer cell lines T-47D, HCC38, human melanoma cell lines A-375 and WM-115 were purchased from iCell (Shanghai, China). Human melanoma cell line MeWo was purchased from FENGHUISHENGWU (Hunan, China). Human melanoma cell line SK-MEL-3 was purchased from COBIER (Jiangsu, China). The human monocyte cell line THP-1 was purchased from the Chinese Academy of Sciences cell bank. The mouse breast cancer cell line 4T1 and mouse melanoma cell line B16-F10 were purchased from Procell Life Science&Technology Co.,Ltd (Wuhan, China). mGSC is a primary spontaneous mouse glioma cell line constructed by our group.

Authentication

STR analysis was used for cell line authentication.

Mycoplasma contamination

Mycoplasma contamination was tested using EZ-PCR Mycoplasma Detection Kit (Biological Industries, 20-700-20)

Commonly misidentified lines
(See ICLAC register)

Name any commonly misidentified cell lines used in the study and provide a rationale for their use.

Animals and other research organisms

Policy information about studies involving animals; ARRIVE guidelines recommended for reporting animal research, and Sex and Gender in Research

Laboratory animals

C57BL/6N (male, 6-8 weeks, Beijing Vital River Laboratory Animal Technology, 213), BALB/c (female, 6-8 weeks, Beijing Vital River Laboratory Animal Technology, 211).

Wild animals

The study did not involve wild animals.

Reporting on sex

For glioma and melanoma analysis, male mice were used. For breast cancer analysis, female mice were used.

Field-collected samples

The study did not involve field-collected samples.

Ethics oversight

The experimental protocol was approved by the ethics committee of The First Hospital of China Medical University. Collection of tumor tissue and clinicopathologic information was obtained with informed consent. Animal experiments were conducted in accordance with the China Medical University Animal Care and Use Committee guidelines and approved by the Institutional Review Board of the First Hospital of China Medical University.

Note that full information on the approval of the study protocol must also be provided in the manuscript.

Clinical data

Policy information about clinical studies

All manuscripts should comply with the ICMJE guidelines for publication of clinical research and a completed CONSORT checklist must be included with all submissions.

Clinical trial registration

NA

Study protocol	The experimental protocol was approved by the ethics committee of The First Hospital of China Medical University. Collection of tumor tissue and clinicopathologic information was obtained with informed consent.
Data collection	Histologically confirmed glioma tissue samples were acquired from The First Hospital of China Medical University with the assistance of neuronavigation between October 10, 2020 and August 18, 2022.
Outcomes	19 glioma samples with sufficient tissue block were sent for RNA seq and immunohistochemistry staining.
